# Exploring morphological aspects, cuticle size and volatile compounds in the fruits of four olive cultivars as possibly interdependent components of *Bactrocera oleae* tolerance

**DOI:** 10.3389/fpls.2025.1681993

**Published:** 2025-11-28

**Authors:** Cosimo Taiti, Luca Lombardo, Gianluca Godino, Luciana Renna, Elisa Masi, Elettra Marone, Marta Beccaluva, Annamaria Ienco, Samanta Zelasco, Santina Rizzo, Enzo Perri, Marianna Rizzo, Pierluigi Rizzo, Veronica Vizzarri, Giovanni Spinelli, Piero Fiorino, Stefano Mancuso, Giovanni Stefano

**Affiliations:** 1 Department of Agricultural, Food, Environmental and Forestry Sciences and Technologies (DAGRI), University of Florence, Sesto Fiorentino, Italy; 2 Council for Agricultural Research and Economics, Reasearch Centre for Olive, Fruit and Citrus Crop (CREA-OFA), Rende, Italy; 3 Department of Bioscience and Agro-Food and Environmental Technology, University of Teramo, Teramo, Italy; 4 Foundation for the Future of Cities, (FFC), Florence, Italy; 5 Department of Biology, Università di Firenze, Florence, Italy

**Keywords:** *Olea europaea* cultivars, olive fly oviposition preference, VOCs, fruit cuticle thickness, Nile Red, *Bactrocera oleae*

## Abstract

*Olea europaea L*. subsp. *europaea*, var. *europaea*, plays a crucial role in cultural identity and economic prosperity across many regions of the Mediterranean Basin. The olive fruit fly, *Bactrocera oleae*, represents a major global challenge to olive and olive oil production. Its larvae feed exclusively on olive fruits, causing severe crop damage and substantial economic losses. In this study, we examined four olive cultivars differing in susceptibility to *B. oleae*, focusing on fruit morphology (weight, maturity index, penetration resistance), cuticle characteristics, and volatile organic compound (VOC) emissions. Confocal microscopy with Nile Red staining was used to analyze cuticle structure, while VOCs were measured using proton-transfer-reaction time-of-flight mass spectrometry (PTR-ToF-MS). Thicker cuticles were associated with reduced infestation, suggesting a mechanical barrier function against oviposition or larval penetration. PTR-ToF-MS analysis revealed cultivar- and ripening stage–specific VOC emission patterns, with certain compounds potentially acting as deterrents or attractants to the olive fly. These results indicate that fruit morphology, cuticle development, and VOC profiles act as interdependent determinants of cultivar-specific tolerance to B. oleae. The integration of these physical and chemical traits provides valuable markers for breeding programs and contributes to the development of sustainable, integrated pest management strategies in olive cultivation.

## 1 Introduction

*Olea europaea* (subsp*. europaea*, var. europaea) (Green, 2002) represents the foundation of both cultural heritage and economic development across Mediterranean and other olive-growing regions. Domestication and selection processes have resulted in more productive and adaptable trees, leading to a constantly increasing number of varieties, with more than 2,000 cultivars spread worldwide (Green, 2002). Italy stands out with the highest level of olive tree biodiversity, boasting approximately 600 described cultivars (Bartolini et al., 2014). The economic significance of olive cultivation is intrinsically linked to tree health and fruit production, which directly impacts both the yield and quality of olive oil.

A primary threat to global olive and oil production, however, is the olive fruit fly, *Bactrocera oleae*. Historically recognized as a major pest in Mediterranean olive-growing regions, this species has expanded its distribution due to globalization and climate change. The larvae of *B. oleae* feed exclusively on olive fruits, posing a severe threat to both intensive and traditional cultivation systems (Grasso et al., 2017). Under favorable climatic conditions, infestations cause substantial crop losses and economic damage to olive farmers.

However, the diverse genetic makeup of olive cultivars offers a potential solution, as some varieties exhibit natural tolerance to this pest. This genetic variability translates into different levels of resistance to *B. oleae* among cultivars (Grasso et al., 2017; Medjkouh et al., 2018). The degree of tolerance to infestation is largely determined by the cultivar’s genetic background, which regulates the activation of specific defense mechanisms. Additionally, *B. oleae* demonstrates a preference for laying eggs on certain olive cultivars over others (Malheiro et al., 2016). Fruit characteristics such as chemical and volatile compound profiles, morphological traits, and ripening stage significantly influence the insect’s behavior and infestation dynamics (Rodríguez et al., 2013; Giunti et al., 2020).

Interactions between the olive tree and its biotic stressors likely shape the compositional traits of the drupes, either reinforcing or diminishing certain features under complex genetic control. For example, the olive tree has developed physical defense barriers, such as a thicker and more elastic fruit skin, which can hinder fruit penetration and subsequent oviposition by pests (Corrado et al., 2023) and chemical defense mechanisms such as the emission of VOCs directly from the olive fruit, which affect the olive fly behavior (Kokkari et al., 2024).

Indeed, before laying eggs, female flies make exploratory punctures (sterile stings) in the olive fruit to determine if the fruit is suitable to host and nourish their developing larvae (Gonçalves et al., 2012). If this test proves favorable, the fly then creates an oviposition chamber by piercing the fruit and depositing an egg within a characteristic triangular incision measuring 1–1.5 mm in length (Rotondi et al., 2021). Daane and Johnson (2010) reported that different cultivars exposed to olive fruit fly showed difference in sterile punctures and larval infestation percentage. Thus, the female is guided both by visual and chemical stimuli in its search for suitable olives, showing preference for (1) large and unripe (green) fruits; (2) fruits with thinner skin (Gonçalves et al., 2012); and (3) cultivars with a specific chemical and volatile emission (Aluja and Mangan, 2008; Daane and Johnson, 2010; Malheiro et al., 2013). The varied capacity of different olive cultivars to cope with *B. oleae* highlights the need to evaluate olive genotypes under diverse environmental conditions and emphasizes the necessity for ongoing research to mitigate the damaging impact of this pest.

This study investigates three potentially interconnected mechanisms that contribute to olive fruit tolerance against *B. oleae*: cultivar morphological diversity, cuticle thickness, and volatile organic compound (VOC) profiling. Among the various traits distinguishing olive cultivars, fruit morphology and biochemical composition are central to determining their susceptibility or tolerance to pests. Specifically, the olive fruit cuticle plays an essential role in safeguarding the fruit from pathogens and pest attacks (Martin and Rose, 2014). Composed of a complex blend of biopolymers, this structure acts as a formidable physical barrier against water loss, pathogen invasion, and mechanical damage. Understanding the composition and dynamics of the olive fruit cuticle may offer new perspectives for pest and disease management strategies. In this context, the interaction between the cuticle and VOC emission remains underexplored. VOCs are lipophilic bioactive molecules involved in plant communication and defense, exerting direct deterrent or toxic effects on herbivores, or indirectly attracting their natural enemies or parasitoids (Heil, 2014; Wang and Erb, 2022). For example, studies on Petunia hybrida have shown that changes in cuticle thickness can influence VOC diffusion and internal redistribution (Liao et al., 2020). Similarly, the oxidation of unsaturated cuticular waxes observed in *Populus trichocarpa*, *P. balsamifera*, and *Zea mays* has been identified as a source of aldehyde volatiles that may trigger plant-to-plant signaling and defense responses (Chen et al., 2023). This research aims to contribute to a deeper scientific understanding of cultivated olives, contributing to the development of a more sustainable and resilient olive production system.

## 2 Materials and methods

Drupes from four olive cultivars (Arbequina, Canino, Coratina, and Nocellara messinese), were morphologically characterized, tested for their degree of susceptibility to *B. oleae*, subjected to VOCs analysis after stings simulation and to Nile Red staining. Fruit sampling was carried out by handpicking during the 2023 olive season at the CREA OFA’s International Olive World Germplasm bank (OWGB; Crosia, Italy, 39° 36’ 54.1’’, 16° 46’ 11.0’’). The study involved three trees per cultivar. Sixty drupes from each tree and cultivars were sampled at complete pit hardening (T1), when fruits become susceptible to *B. oleae* oviposition (Baratella et al., 2017), and at drupe veraison when olives are typically harvested (T2). The cultivars were chosen based on preliminary observations in the collection field that suggested varying levels of susceptibility to the olive fruit fly, these initial findings required further confirmation through this study.

### 2.1 Olives morphological aspects and *B. oleae* infestation level

A subsample of forty drupes/tree (120 drupes per cv and sampling date) was used to evaluate the ripening stage according to the Jaén index (IOC, 2011) and average drupe weight, as well as to assess the level of susceptibility to *B. oleae*. This assessment was based on the presence of sterile punctures, all preimaginal stages of the olive fruit fly (alive or dead), and abandoned tunnels.

Additionally, active infestation (presence of eggs, live first and second instar larvae) and harmful infestation (live third instar larvae, pupae, and abandoned tunnels) were evaluated after destructive analyses under a binocular microscope following Vizzarri et al. (2023) along with dead infestation (dead first, second, and third instar larvae, and pupae) and infestation limited to sterile punctures (dead first, second, and third instar larvae, and pupae) and infestation limited to sterile punctures. Furthermore, 15 drupes per tree, cultivar, and sampling date were collected to measure penetration resistance (PR) using a Fg-5000A electronic force gauge penetrometer (Lutron Electronic Enterprise Co., Ltd., Taipei, Taiwan) equipped with a 1-mm tip. Each fruit was pierced three times along the equatorial diameter.

### 2.2 Nile Red staining and tissue analysis by confocal microscopy

For the analysis of lipid accumulation within the olive fruit tissue, Nile Red staining was employed, followed by confocal microscopy. Nile Red (Sigma-Aldrich) was prepared as a stock solution at a concentration of 1 mg/mL in DMSO, and then aliquoted into 1.5 ml tubes to minimize thawing cycles and preserve its stability. A thin fruit slice was carefully obtained using a razor blade. Each slice was then incubated with the Nile Red solution (2ul of Nile Red at a concentration of 1 mg/mL and 998 ul of milliQ water) for 5 minutes.

After staining, samples were observed using a Leica SP5 confocal microscope equipped with a 40x objective (HCX PL APO OIL UV). For imaging the blue-shifted excitation of Nile Red, a 458 nm laser line was used, and the fluorescence emission signal was acquired between 475–495 nm. Three olive drupes for each cultivar were used and three experimental replicates were conducted on each of the two sampling dates to ensure the robustness and reliability of the results presented in this study.

### 2.3 Stings simulation and VOC analysis

Proton Transfer Reaction Time-of-Flight Mass Spectrometry (PTR-ToF-MS) was employed to monitor the emission of volatile organic compounds (VOCs) from the fruits of the four distinct olive cultivars. The analysis was conducted under controlled climatic conditions at 25 ± 1°C and 45–55% relative humidity using a PTR-MS 8000 instrument (Ionicon Analytik GmbH, Innsbruck, Austria). The tool was operated in its standard configuration with H_3_O^+^ as a reagent ion. The following settings were applied: drift tube voltage of 600 V, drift tube temperature 60°C and drift pressure 2.2 mbar, E/N of 145 Td, drift gas flow of 40 sccm, and a mass scan range of m/z 13–250. The internal calibration of the PTR-ToF spectra was performed offline using the following signals: m/z 29.9974 (NO), m/z 59.049 (C_2_H_5_O_2_), and m/z 180.937 (C_6_H_4_Cl_3_). To ensure high mass accuracy, we followed the procedure described by Cappellin et al. (2010), which employs a three-point calibration. This approach enabled high mass accuracy across the relevant mass range, sufficient for molecular formula identification. After mass peak selection and extraction, tentative compound identification was performed based on the molecular formulas provided by the analysis software and compared with data reported in the literature (Brilli et al., 2011; Rasulov et al., 2019).

With the aim of simulating the fly attacks, prior to analysis, fruits were stung using an Insulin pen with a 1.5 mm long and 0.3 mm diameter needle to simulate and replicate the ovipositor of *Bactrocera oleae* in both penetration depth and puncture diameter. Specifically, four stings were done at the opposite side of each fruit. For VOC collection, 10 stung fruits per cultivar were placed in 2/3 L glass bottles and incubated for 5 minutes. After incubation, headspace VOCs were analyzed following the experimental design and analytical methodology described by Taiti et al. (2022). Samples were analyzed in randomized order, with a 5-minute interval between runs to flush the inlet tubing and minimize memory effects. A Zero Air Generator (Peak Scientific, Inchinnan, UK) was used to purge the inlet line between measurements. In total, 16 PTR-ToF runs were conducted (4 cultivars x 2 sampling times x 2 replicates). Raw data (expressed as counts per second, cps) were acquired with TofDaq software v. 183 (Tofwerk AG, Innsbruck, Switzerland) using a dead time of 20 ns for the Poisson correction. Data were subsequently converted to parts per billion by volume (ppbv). After removal of NO^+^, O_2_^+^, and water clusters ions, a threshold of 0.5 ppbv was applied; all compounds below this threshold were excluded from further analysis. To ensure comparability of VOC profiles across cultivars despite differences in fruit mass, all data were normalized to a standard fruit weight of 100g. Headspace analyses were repeated for each sampling time using the same protocol.

### 2.4 Statistics

**Figure 1:**
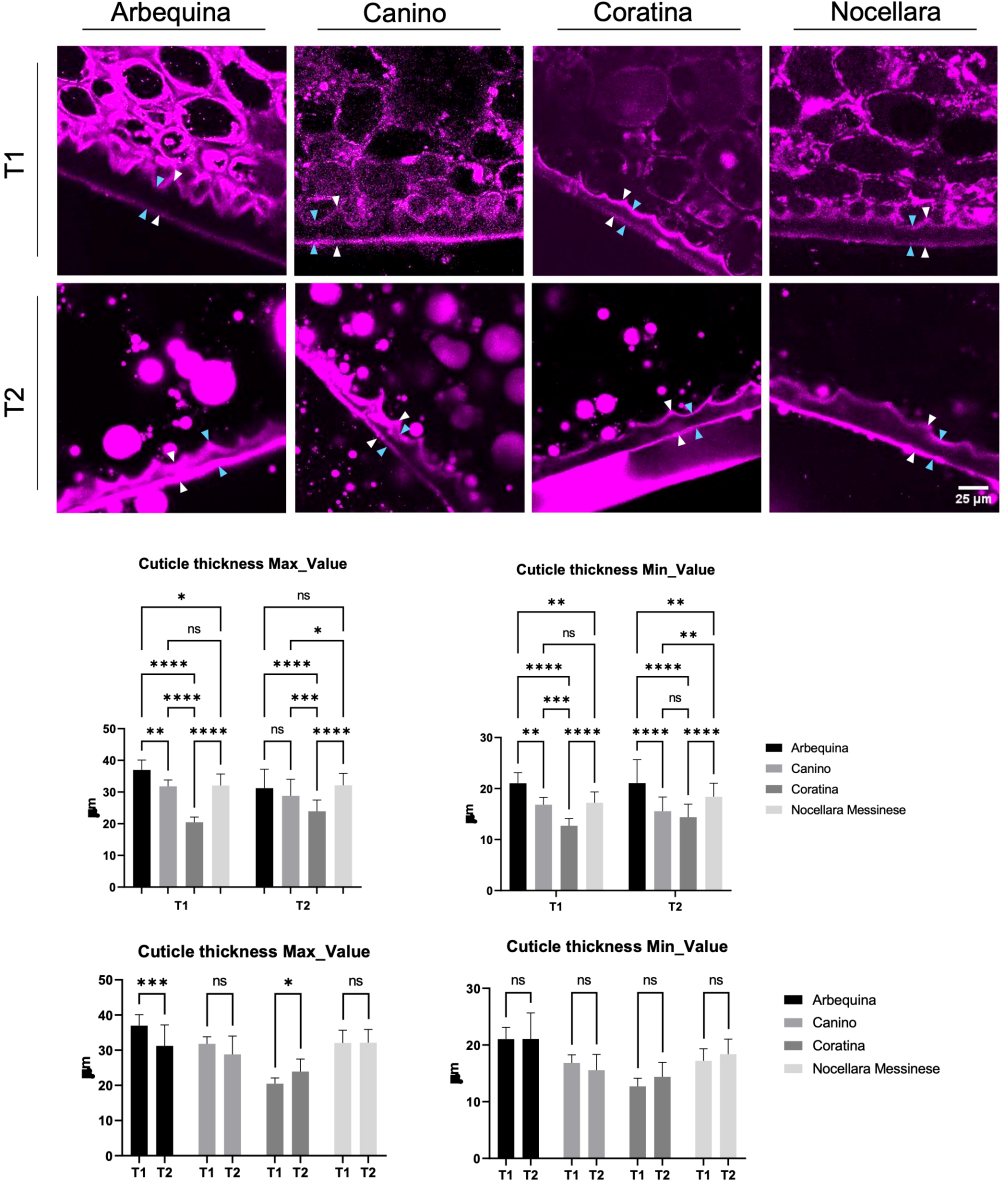
Confocal images of arbequina, canino, coratina and nocellara drupe cuticle labeled with Nile Red. White arrowheads indicate the points where the maximum cuticle thickness was measured. Cyan arrowheads indicate the points where the minimum cuticle thickness was measured. Minimum and maximum cuticle value for each sample monitored during two phases of fruit maturation (early and late in the development). n.s., not significant; *P  <  0.05, **P  <  0.01, ***P  <  0.001, ****P  <  0.0001. Error bars represent the S.E.M. Cuticular layer that enveloped the fruit surface at the two different developmental stages. n.s., not significant; *P  <  0.05, **P  <  0.01, ***P  <  0.001, ****P  <  0.0001. Error bars represent the SEM.

One-way ANOVA analysis was employed in conjunction with Tukey’s HSD post-tests to analyze both the morphological (fruit weight, penetration resistance and Jaén index) and *B. oleae* infestation data, of the tested cultivars at the two sampling dates. Capital letters A, B, C =P <0.01; lowercase letters a, b, c, =P <0.05. (**Tables 1**, **2**) Pearson product-moment correlation coefficients between the 21 protonated masses emitted by the fruits and wounding punctures, sterile punctures, and sound olives, respectively, are calculated for each individual VOC. Computations were performed by Stat graphics Centurion 19.6.05. A Factor Analysis (FA) was applied to highlight the relationships among the 4 cultivar olive fruit samples collected at T1 and T2, respectively, considering as factors the three levels of infestation (sound olives, sterile punctures, and wounding punctures) and the protonated m/z having Pearson product-moment correlation coefficients (P) ≥-0.5 or ≥0.5, respectively. Computations were performed by XLSTAT 2024.4.1.1425. Cuticle thickness difference (graphs in **Figure 1**) where evaluated by One-way ANOVA, Sidak’s multiple comparison test. Using asterisks to better compare each couple of cvs. Asterisks denote significance in statistical analysis: n.s.: not significant; *P  <  0.05, **P  <  0.01, ***P  <  0.001, ****P  <  0.0001.

**Table 1:** Comparison of fruit weight, penetration resistance (PR), and Jaén index among cultivars at two sampling dates.

CV	First sampling time (T1)			Second sampling time (T2)		
	Drupe wei4ght fw (g)	PR (N)	Jaén Index	Drupe weight fw (g)	PR (N)	Jaén Index
Arbequina	0.81 ± 0.12 C	4.76 ± 2.31 b	1.00 ± 0.00	1.79 ± 0.27 C	3.14 ± 1.73 ab	2.46 ± 0.26
Canino	0.77 ± 0.12 C	3.37 ± 1.78 b	0.70 ± 0.03	1.21 ± 0.29 C	2.51 ± 0.65 b	2.54 ± 0.31
Coratina	1.64 ± 0.36 B	6.31 ± 3.05 b	0.60 ± 0.00	3.18 ± 0.86 B	2.53 ± 0.66 b	2.12 ± 0.45
Nocellara messinese	4.15 ± 1.09 A	9.22 ± 4.85 a	0.60 ± 0.57	5.03 ± 1.51 A	3.59 ± 0.88 a	2.42 ± 1.10

Fruit weight, penetration resistance (PR) and Jaén index of the tested cultivars at the two sampling dates. Different uppercase and lowercase letters indicate statistical significances at 99% and 95% levels, respectively, through *post hoc* Tukey’s HSD test. PR, Penetration resistance.

**Table 2:** Percentage of *B. oleae* infestation.

First sampling time							
CV	Total infestation (%)	Active infestation (%)	Harmful infestation (%)	Dead infestation (%)	Infestation limited to sterile punctures/Total infestation (%)	Wounding punctures (%)	Sterile punctures (%)
Arbequina	14.00 ± 2.31 A	0.00 ± 0.00	0.00 ± 0.00	0.00 ± 0.00	100 ± 0.00	0	14
Canino	20.00 ± 2.00 A	0.00 ± 0.00	0.00 ± 0.00	0.00 ± 0.00	100 ± 0.00	0	20
Coratina	54.00 ± 14.14 B	0.00 ± 0.00	6.00 ± 2.84	20.00 ± 11.31	64.49 ± 11.65	26	28
Nocellara messinese	72.00 ± 2.00 B	0.00 ± 0.00	0.00 ± 0.00	10.00 ± 8.48	86.11± 11.79	10	62
Second Sampling time							
CV	Total infestation (%)	Active infestation (%)	Harmful infestation (%)	Dead infestation (%)	Infestation limited to sterile punctures/Total infestation (%)	Wounding punctures (%)	Sterile punctures (n.)
Arbequina	36.00 ± 4.00 a	1.33 ± 0.31	0.00 ± 0.00	4.00 ± 1.00	86.30 ± 15.17 A	5	31
Canino	40.00 ± 8.49 a	0.00 ± 0.00	12.00 ± 2.83	2.00 ± 1.01	62.50 ± 17.68 AB	14	26
Coratina	80.00 ± 16.97 b	20.00 ± 8.49	32.00 ± 11.31	14.00 ± 5.66	32.23 ± 12.28 B	66	14
Nocellara messinese	76.00 ± 28.29 b	0.00 ± 0.00	6.00 ± 2.83	26.00 ± 11.79	62.80 ± 12.21 AB	32	44

Percentage of *B. oleae* infestation of the considered cultivars at the two sampling dates. The last two columns report the calculated wounding punctures (active +harmful+dead infestation), and sterile punctures (100-wounding punctures+sound olives) respectively. Different uppercase and lowercase letters indicate statistical significances at 99% and 95% levels, respectively, through *post hoc* Tukey’s HSD test”.

## 3 Results

### 3.1 Olives morphological aspects and *B. oleae* infestation level

The results of the analyzed traits showed, in general, considerable variability (**Table 1**) over time among cultivars, as well as intra-cultivar variability in the case of PR (penetration resistance). A strong direct correlation (Pearson’s r=0.96) was found between drupe weight and PR at the early stage of maturation, with the cv Nocellara messinese presenting the statistically significant highest values at both sampling dates. Conversely, the values obtained from the fruits of Arbequina and Canino were overall very similar. The notable increase in weight recorded between the two sampling dates was counterbalanced by an equally remarkable reduction in PR. The cvs Arbequina and Coratina showed the highest weight gain (+120.99% and +93.90%, respectively; compared to +57.143% and +21.21% for the cvs Canino and Nocellara messinese), while drupe firmness was more pronouncedly reduced in cv Nocellara messinese (-61.150%) and Coratina (-59.89%). Nevertheless, the drupes of Nocellara messinese still displayed the highest PR at T2 with an average of 3.59 Newton. Drupe color ranged from green to yellowish green at T1 and from yellowish green to purple (veraisoned drupes) at T2.

Regarding *B. oleae* infestation level of the tested cultivars (**Table 2**), fruits of Arbequina and Canino resulted to be the less infested both at T1 (86 and 80% of non-infested drupes, respectively) and T2 (64 and 60%), while the drupes of Nocellara messinese and Coratina at the two sampling times recorded the highest statistically significant percentages of overall infestation (72 and 76% vs 54 and 80%, respectively). In particular, at T1, total infestation for the cv Arbequina and Canino was only represented by sterile punctures, whose percentages remained quite high even at T2 (86.30 and 62.50% of total infestation, respectively). The sum of sterile punctures (for the most part) and dead infestation made up 100% of total infestation in the drupes of the cv Nocellara messinese at T1 and 92% at T2. A further different trend was displayed by the drupes of Coratina which suffered the highest active and harmful infestation.

### 3.2 Nile red to screen for cuticle size in different cultivars during fruit maturation

Following Nile Red staining, olive drupe samples from all cultivars exhibited fluorescence due to the dye’s ability to bind to hydrophobic structures, such as the cuticle. This fluorescent dye has been previously utilized to investigate cuticle dynamics in plant tissues, demonstrating its effective absorption by the cuticle. Fluorescence microscopy revealed distinct staining patterns that corresponded to cuticle localization, allowing for the determination of minimum and maximum cuticle thickness values for each sample across two key phases of fruit maturation: early and late development. The intensity of fluorescence varied among cultivars (**Figure 1**), directly reflecting differences in cuticle thickness. Early in development, Arbequina, Canino, and Nocellara Messinese displayed the highest fluorescence signals for maximum cuticle thickness compared to Coratina (**Figure 1**). Similar differences were also observed for minimum cuticle thickness, except for Canino, which resembled Nocellara Messinese in this regard. This general profile was maintained at later developmental stages (**Figure 1**), and statistical analysis confirmed significant variations in cuticle size among the cultivars (**Figure 1**). To further visualize and monitor changes in cuticle size throughout fruit maturity, Nile Red staining was again applied to fruit sections of Arbequina, Canino, Coratina, and Nocellara Messinese. Fluorescence patterns associated with the cuticular layer dynamically changed across the two developmental phases, as determined by fluorescence microscopy. Notably, the fluorescence signal relative to the maximum cuticle thickness in Arbequina was observed to decrease during the maturation process. Conversely, in Coratina, the presence of a cuticular layer enveloping the fruit surface increased as the fruit underwent ripening. For the remaining cultivars, no significant difference was observed between the two distinct developmental stages (**Figure 1**). Similarly, contrasting trends in cuticle thickness during veraison and full ripening have been reported in nine olive cultivars in Spain (Diarte et al., 2019), with the ‘Arbequina’ cultivar showing a slight decrease under irrigated conditions and a more pronounced decrease in rainfed orchards (Diarte et al., 2023). Overall, our data fall within the range reported for other olive cultivars in studies employing scanning electron microscopy (SEM) (Lanza et al., 2015; Goldental-Cohen et al., 2019), as well as in studies where cuticle thickness was quantified using light microscopy combined with toluidine blue (Gomes et al., 2012) or Sudan IV staining (Diarte et al., 2023).

### 3.3 VOCS profile in olive fruits during early and late developmental stage

Using PTR-ToF-MS we identified different compounds emitted from olive fruits, all of which have been previously reported in other studies. The VOC data obtained are reported in **Table 3**. Qualitative differences in VOC composition were observed among the four olive cultivars and between the two sampling times. Comparison of the data (**Table 3**) revealed changes in VOC emissions from the artificial bite made on the olive fruits. Olives respond differently to internal physiological changes during fruit maturation: some volatile organic compounds (VOCs) like C5 and C6 decreased, while others, such as methanol and acetaldehyde, increased in release intensity across all cultivars and both sampling times. As suggested by White et al. (2016) in mango fruits, the increase in methanol emission could be due to the higher methyl esterification of pectins when fruits are immature compared to when they are ripe. Accordingly, the more marked increase in methanol release recorded with the progression of fruit maturation in the cvs Nocellara messinese (+152.01%) and Coratina (+141.09%) compared to Arbequina (+43.69%) and Canino (+15.43%) is accompanied by a specular reduction in drupe penetration resistance (-61.06 and -59.90% vs -34.03 and -25.52%, respectively). Acetaldehyde is a naturally occurring metabolite, resulting from pyruvate decarboxylation, which can be reduced to ethanol. As reported by Pesis (2005), both climacteric and non-climacteric fruit produce a lot of acetaldehydes mainly depending on their genetic characteristics. Acetaldehyde and ethanol which are precursors of natural aroma, are typically produced because of the normal ripening process (El Hadi et al., 2013). Methanol, ethanol, and acetaldehyde emissions increased throughout the maturation process, depending on the cultivar. Similarly, total VOC emissions increased for each cultivar between the two sampling points. These results align with findings by Beltrán et al. (2015) who reported variations in ethanol emissions among olive cultivars and at different maturation stages. For example, ethanol levels were lower in Arbequina and Canino, average in Coratina, and higher in Nocellara Messinese at both time points (see **Table 3**). Additionally, each cultivar exhibited different rates of increase in ethanol emissions depending on its ripening stage. Total VOC emission intensity also varied by cultivar and sampling time. Nocellara Messinese consistently showed the lowest overall emission intensity at both time points (**Table 3**). Moreover, an increase in dimethyl sulfide emissions was observed at T2 (approximately 10-fold in Nocellara Messinese, 6-fold in Coratina, and 4-fold in Arbequina). This increase could be related to the enzymatic activity of yeasts naturally present on the olive carposphere (Guerrini et al., 2019) and may also be linked to *B. oleae* preferences (Brkić Bubola et al., 2023). The rise in yeast and/or bacterial populations on the fruit surface may also contribute, at least in part, to the increased ethanol emissions observed in veraison-stage drupes. On the contrary, the emission intensity of most C5 and C6 compounds declined during ripening. Notably, signals detected at m/z 69.069 (Tentatively Identified as isoprene), 81.069 (TI C6 fragments), 99.080 (TI (E)-2-hexenal), and 101.059 (TI (Z)-3-hexen-1-ol/hexanal) all decreased between T1 and T2 across all cultivars. This trend is consistent with the known reduction in LOX (lipoxygenase) and hydroperoxide lyase (HPL) activities during fruit ripening (Yilmaz et al., 2002). An exception to this trend is the signal at m/z 87.080 (TI as pentanal/3-methylbutanal, aldehydes associated with fermentative processes), which tended to increase. At both sampling times, Nocellara Messinese and Coratina displayed the lowest emission of C5 and C6 compounds. This general C5/C6 decline (which imparts green-fruity notes), coupled with an increase in dimethyl sulfide, acetaldehyde, propanal, and acetates (which contribute to odor defects), represents an important factor when determining the optimal harvesting period (Yan et al., 2020; Taiti et al., 2022).

**Table 3:** Identification and emission intensity of volatile organic compounds (VOCs) detected in olive cultivars by PTR–MS.

N° compounds	M/Z	[MH]+	TI	First sampling time				Second sampling time			
				Noc.	Cor.	Arb.	Can.	Noc.	Cor.	Arb.	Can.
1	31.018	CHO_3_^+^	Formaldehyde	19.10	17.63	24.94	20.82	18.71	20.57	18.62	23.88
2	33.033	CH_5_O^+^	Methanol	778.4	1047.20	2012	2143.56	1961.64	2524.73	2891.08	2474.31
3	41.038	C_3_H_5_^+^	Fragments (alcohols and ester)	10.45	13.52	12.51	17.57	12.80	13.69	9.75	12.32
4	43.018	C_2_H_3_O^+^	Fragments (ester)	23.04	30.75	23.68	28.29	41.34	54.68	20.16	41.79
5	43.054	C_3_H_7_^+^	Fragments (alcohols)	3.44	4.69	4.41	4.78	7.42	9.03	5.32	7.46
6	45.033	C_2_H_5_O^+^	Acetaldehyde	356.45	364.5	638.50	891.82	401.45	509.54	896.88	960.81
7	47.049	C_2_H_7_O^+^	Ethanol	3.08	2.64	1.93	1.81	4.85	3.56	1.95	2.26
8	55.055	C_4_H_7_^+^	C4 aldehydes fragment	8.36	10.08	8.69	13.10	19.63	15.71	8.35	14.97
9	57.033	CH_3_O_5_^+^	Fragments (hexanal/hexyl acetate)	2.87	6.56	3.91	6.57	7.51	10.85	2.47	11.12
10	57.069	C_4_H_9_^+^	Hexanol fragment	2.00	2.77	2.16	2.70	3.98	7.04	5.02	4.92
11	59.050	C_3_H_7_O^+^	Propanal, Acetone	20.52	30.40	32.36	28.32	48.37	48.97	47.16	46.86
12	61.027	C_2_H_5_O_2_^+^	Acetates	12.19	19.50	18.71	18.28	34.21	28.99	12.89	23.26
13	63.027	C_2_H_7_S^+^	Dimethyl sulphide	1.22	1.00	1.25	1.28	12.54	6.25	5.52	1.23
14	69.069	C_5_H_9_^+^	Isoprene	2.90	5.36	7.74	7.39	2.47	4.43	4.33	5.50
15	73.065	C_4_H_9_O^+^	Isobutanal/ butanone	2.37	2.57	2.54	2.59	4.95	5.60	6.76	5.38
16	81.070	C_6_H_9_^+^	C6 compounds	25.09	29.55	39.23	41.51	11.51	17.29	26.25	32.77
17	83.086	C_6_H_11_^+^	C6 compounds/ hexenol	2.52	2.97	3.01	3.97	3.14	3.08	3.39	2.62
18	87.080	C_5_H_11_O^+^	Pentanal/3-methylbutanal	1.41	1.67	1.98	2.35	2.22	3.05	2.85	2.87
19	99.080	C_6_H_11_O^+^	C6 (e.g. Hexenal)	7.29	10.66	15.50	15.96	2.86	4.88	11.43	12.38
20	101.059	C_5_H_9_O_2_^+^	Hexanal/pentanedione	2.50	2.92	4.96	4.51	2.02	3.14	3.58	3.82
21	105.069	C_8_H_9_^+^	Styrene	0.70	1.10	0.52	0.59	0.58	0.66	0.25	0.39
Total C5 emission				6.81	9.94	14.68	14.25	6.71	10.62	10.76	12.18
Total C6 emission				34.90	43.18	57.74	61.44	17.51	25.26	41.06	47.76
Total Vocs emission				1285.9	1617.83	2868.57	3267.64	2614.53	3313.80	3995.2	3701.34

Number of compounds detected, along with their *m/z*, chemical formula, tentative identification (TI), and emission intensity (ppbv). Noc., Nocellara messinese; Cor., Coratina; Arb., Arbequina; Can., Canino. [MH]+ denotes the Protonated Chemical Formulae. TI stands for Tentative Identification. VOCs are Volatile Organic Compounds. TI was performed based on the exact masses provided by the tool and compared with GC–MS data and fragmentation patterns (when available), as well as with PTR–MS literature. Only signals with an abundance greater than 0.50 ppbv were considered. Total C5 emissions were calculated as the sum of *m/z* 69, 87, and 101; total C6 emissions were calculated as the sum of *m/z* 81, 83, and 9.

## 4 Discussions

### 4.1 Morphological traits, cuticle thickness, and VOC emissions in relation to *B. oleae* infestation

**Figure 2:**
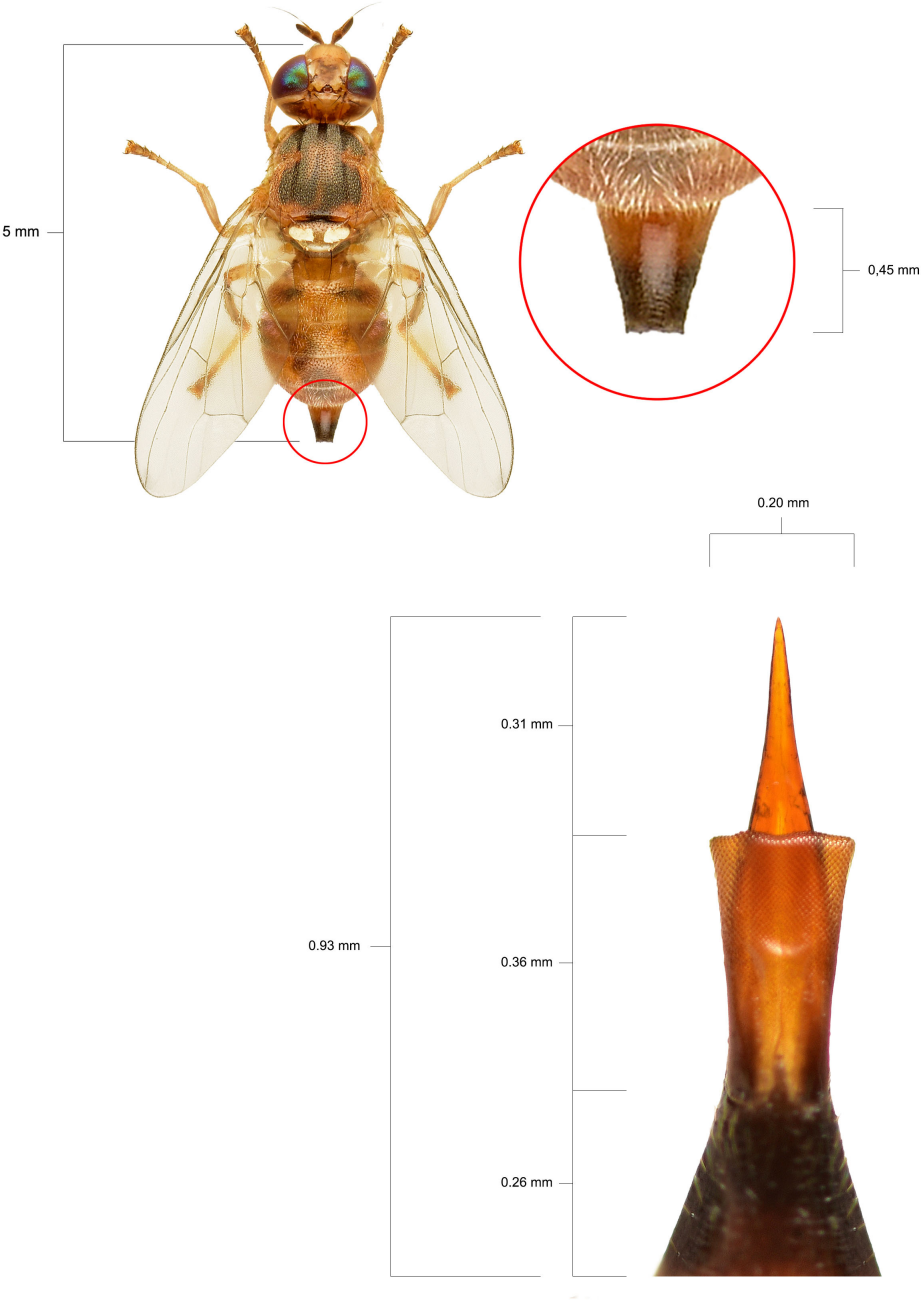
Typical dimensions of a female specimen of *B*. *oleae* and its ovipositor apparatus equipped with a sting.

Our results highlight the multifactorial nature of olive fruit tolerance to *B. oleae* infestation, demonstrating how morphological traits, cuticle structure, and VOCs emissions could collectively influence the susceptibility of different cultivars. Morphologically, a clear relationship was observed between olive drupe characteristics and infestation levels. Cultivars Arbequina and Canino consistently showed the lowest infestation and penetration resistance (PR) values, with minimal reductions in PR between sampling dates (-34.04% and -25.47%, respectively), likely due to the high prevalence of non-infested fruits within these cultivars. On the contrary, Nocellara Messinese exhibited the lowest percentage weight gain and highest percentage decrease in PR, consistent with its earlier fruiting stage at T1 and the physiological softening of fruit during ripening, driven by enzymatic degradation of cell wall polysaccharides (Jimenez et al., 2001; Cappelli et al., 2024). The higher PR values observed in Nocellara Messinese (9.22 N) and Coratina (6.22 N) support previous findings indicating *B. oleae’s* preference for large fruits with high pulp hardness (Gonçalves et al., 2012; Rizzo et al., 2012). It is important to contextualize the PR values reported here within the framework of ovipositor mechanics: the pressure exerted by the olive fly ovipositor ranges from 7.2 to 1.9 N mm^-^², applied over a minuscule surface (~1 × 10^-4^ mm²) (**Figure 2**).

This suggests that measured PR values are indicative rather than absolute resistance parameters. Notably, Coratina and Nocellara Messinese both with the highest total infestation, showed contrasting behaviors: Coratina experienced high levels of active and harmful infestation, whereas Nocellara Messinese displayed a significant prevalence of sterile punctures and dead infestation. This implies a post-puncture defense mechanism in Nocellara Messinese that either impedes successful egg laying or inhibits pre-imaginal development, a phenomenon aligned with previous studies demonstrating induced plant defenses in *B. oleae*-infested olives, including the production of VOCs, phytohormones, and defense proteins (Alagna et al., 2015; Bogka et al., 2023). Moreover, whole-plant VOC emissions are known to affect *B. oleae* mating and oviposition preferences (Malheiro et al., 2016; Kokkari et al., 2021). The slightly lower maturation index in Coratina drupes may also contribute to fly preference for greener fruits (Malheiro et al., 2019). The correlations between VOC profiles and infestation status (see VOC section) further support these defense mechanisms. Regarding cuticle thickness, Nile Red staining combined with confocal scanning microscopy (CSM) revealed significant variability among cultivars, enabling effective screening of this trait beyond laborious methods such as Transmission Electron Microscopy. The thickest cuticle was found in the less susceptible Arbequina cultivar, suggesting that a thicker cuticle serves as a physical barrier to *B. oleae* oviposition. The comparison between Nocellara Messinese (thicker cuticle, mainly sterile infestation) and Coratina (thinner cuticle, higher active infestation) reinforces this hypothesis. Such morphological barriers may reduce the likelihood of ovipositor penetration and successful infestation, indicating cuticle thickness as a promising trait for breeding resistant cultivars. These findings have direct implications for sustainable olive pest management, emphasizing cuticle morphology’s role in infestation susceptibility.

**Figure 3:**
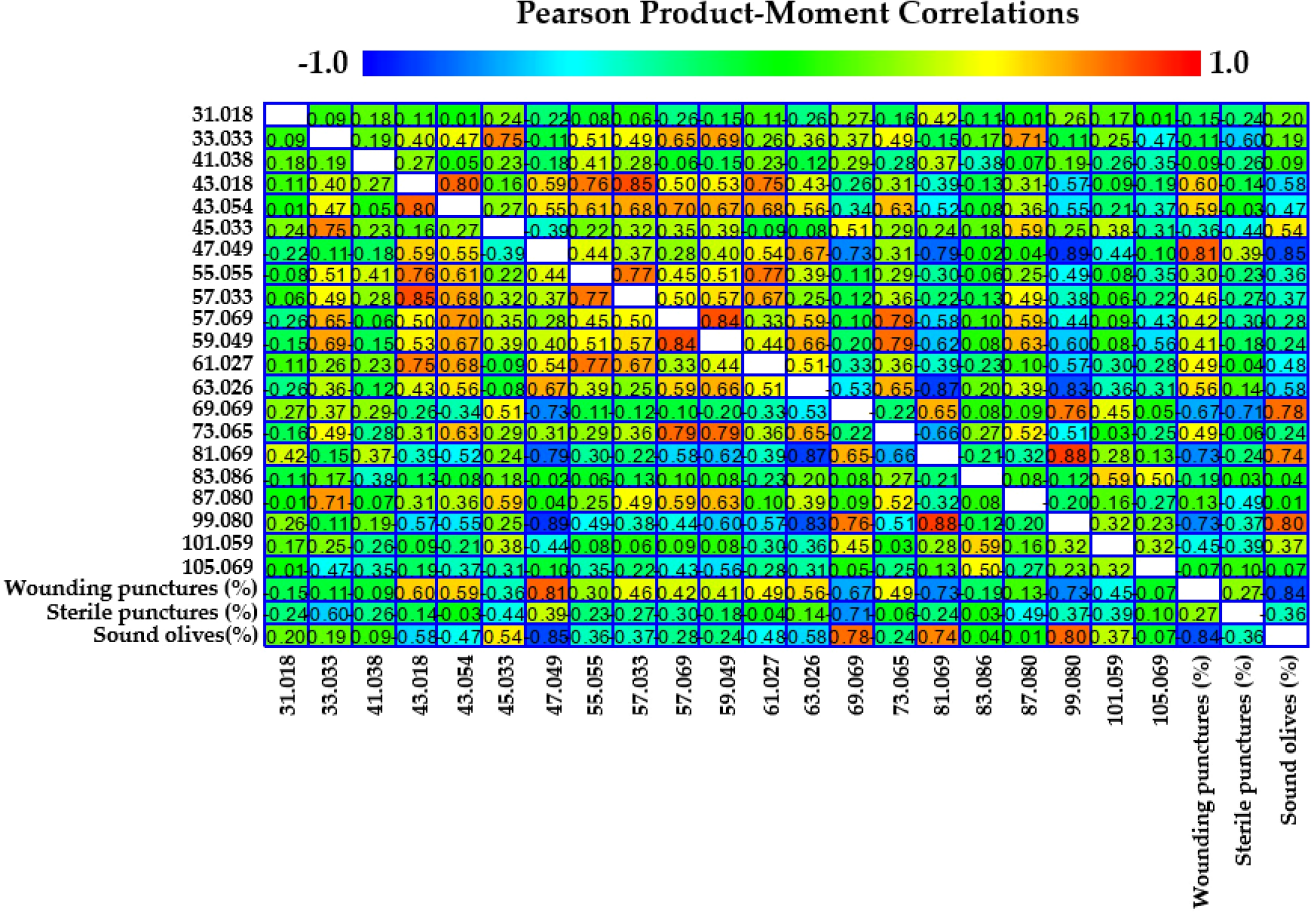
Pearson product-moment correlation coefficients between 21 protonated masses and wounding punctures, sterile punctures, and sound olives. Color is used to denote the magnitude of the correlations, which range from -1 to +1.

VOCs analysis further elucidated the biochemical interactions between olive fruits and *B. oleae.* By the VOC analysis emerged 21 protonated masses emitted by the fruits and their correlations with infestation levels were investigated. In particular, we focused on “wounding punctures,” which include active, harmful, and dead infestations; these punctures represent actual damage caused by larval development inside the fruit. VOC emissions were measured in four olive cultivars at two key ripening stages (T1 and T2). The detailed results of these correlations are presented in **Figure 3** and summarized in **Table 4**.

**Table 4:** Protonated m/z values showing significant correlations.

Protonated m/z	Compounds Tentative Identification	Wounding punctures (%)	Sterile punctures (%)	Sound olives (%)
43.018	Fragments (ester)	0.60		-0.58
43.054	Fragments (alcohols)	0.59		
47.049	Ethanol	0.81		-0.85
69.069	Isoprene	-0.67	-0.71	0.78
81.069	C6 compounds fragments	-0.73		0.74
99.080	C6 (e.g. Hexenal)	-0.73		0.8

Protonated m/z having Pearson product-moment correlation coefficients (r) ≥-0.5 (negative correlation) or ≥0.5 (positive correlation) with the considered variables, and P-values <0.05 (significant non-zero correlations at the 95.0% confidence level).

**Figure 4:**
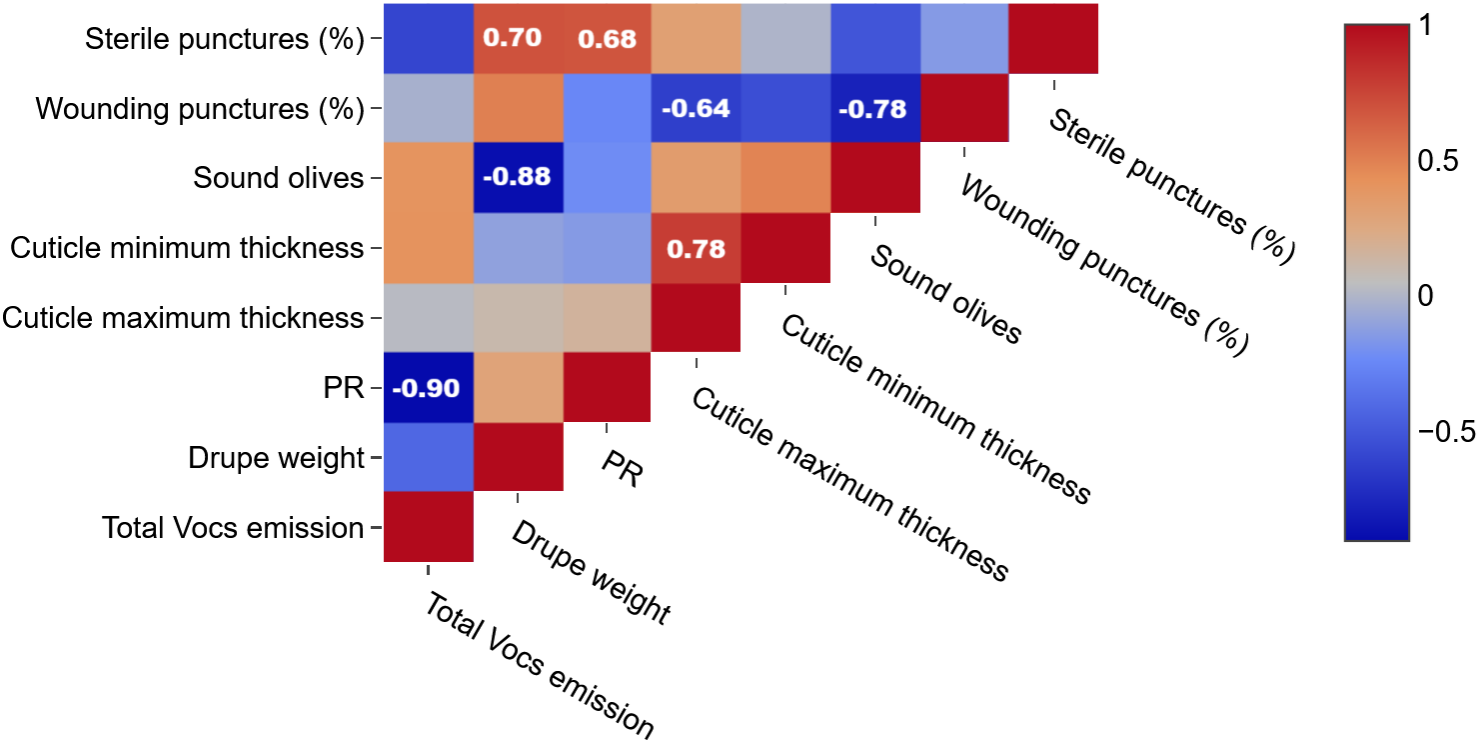
Pearson product-moment correlation coefficients between sterile punctures, wounding puncture e sound olives, cuticle minimum thickness, cuticle maximum thickness, PR, drupe weight, total VOCs emission. Color is used to denote the magnitude of the correlations, which range from -1 to +1. Reported values represent statistically significant (p<0.05) correlations.

**Figure 5:**
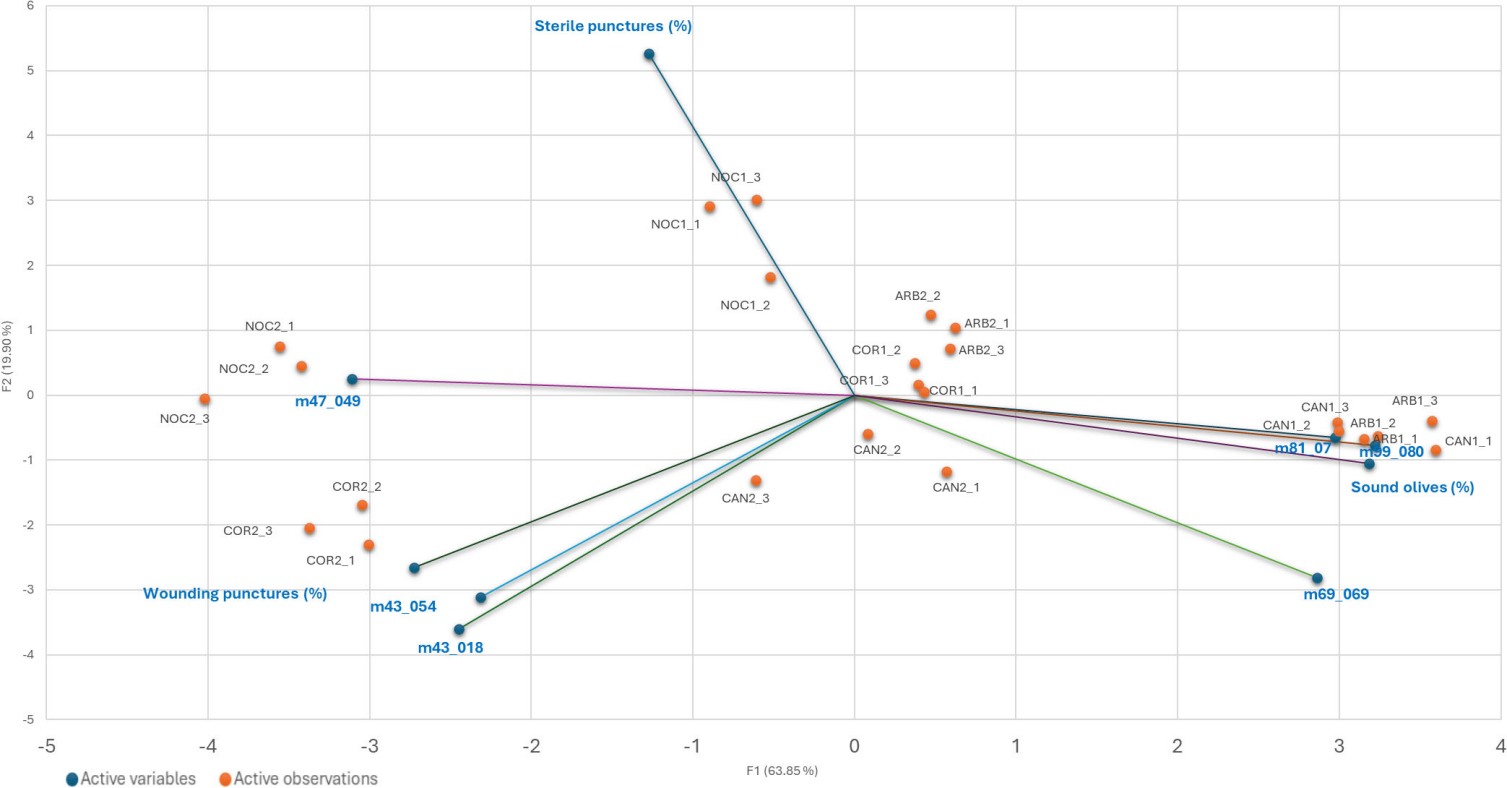
Biplot from Factor analysis. Relationships among the 4 cultivar olive fruit samples collected at T1 and T2, respectively, the three levels of infestation (sound olives, sterile punctures, and wounding punctures) and the protonated m/z having Pearson product-moment correlation coefficients (P) negative (≥-0.5) or positive (≥0.5).

Moreover, the Factor Analysis plot (**Figure 4**) reveals that certain compounds, such as ethanol (m/z 47.049) and fragments corresponding to esters and alcohols (m/z 43.018, 43.054), were positively correlated with the percentage of wounding punctures at both sampling times, indicating their potential roles as attractants. Conversely, C5 and C6 compounds, which are early products of the lipoxygenase (LOX) cascade such as isoprene (m/z 69.070), C6 fragments (m/z 81.070), and hexenal (m/z 99.080) showed strong positive correlations with sound olives and negative correlations with wounded fruits, suggesting a deterrent effect on ovipositing females. These C6 compounds have been documented to disrupt insect behavior by acting as repellents or masking attractant signals (Germinara et al., 2024). Similarly, isoprene, produced via the mevalonic acid pathway, is a well-known general insect repellent emitted by vegetation (Kokkari et al., 2024). The classification of cultivars based on VOC emissions aligns with infestation susceptibility: Arbequina and Canino, which emit higher levels of these deterrent VOCs early in maturation, were classified as tolerant, while Coratina and Nocellara Messinese, with consistently lower levels, were classified as susceptible regardless of their ripening stage as assessed by the Jaén Index stage (**Table 1**). Therefore, Factor Analysis (**Figure 5**) highlights the association between specific VOCs and infestation categories, confirming the dual roles of attractant and deterrent compounds.

Interestingly, no direct correlation was found between cuticle thickness and total VOC emissions (**Figure 4**). However, total VOC emission was negatively correlated with fruit firmness (Pearson’s r = –0.90), while cuticle thickness correlated negatively with harmful infestation levels (r = –0.62) and positively with the proportion of sterile punctures on total infestation (r = 0.68). These data indicate that morphological and biochemical defenses function through partially independent but complementary mechanisms to modulate *B. oleae* infestation dynamics.

## 5 Conclusions

This study provides a comprehensive analysis of the interplay between morphological traits, cuticle thickness, and VOC emissions in olive fruits from four cultivars with differing susceptibility levels to *B. oleae*. Our results highlight how cultivar-specific factors influence olive fruit fly infestation dynamics, with Arbequina and Canino consistently exhibiting lower levels of active infestation, likely due to a combination of higher cuticle thickness, lower pulp hardness, and VOC emission profiles dominated by deterrent compounds such as isoprene and C6 volatiles. Conversely, Nocellara Messinese and Coratina showed higher infestation rates, with Coratina particularly susceptible to active and harmful damage. Interestingly, while Nocellara Messinese exhibited the highest penetration resistance early in maturation, its fly damage was largely limited to sterile punctures and dead infestation, suggesting the presence of post-oviposition defense mechanisms. Cuticle thickness, was negatively associated with harmful infestation levels, supporting the role of this barrier in deterring successful oviposition. Moreover, dynamic changes in cuticle development during maturation varied among cultivars, with Coratina showing a late-stage increase in cuticle thickness possibly as an induced response to biotic stress. The VOC profiling identified methanol, ethanol, and acetaldehyde as key emissions associated with fruit ripening and could be a potential fly attraction, while C5 and C6 volatiles, including hexenal and isoprene, decreased with maturity and were positively associated with fly deterrence. The higher concentrations of VOCs such as isoprene and hexenal observed in tolerant cultivars may indicate a potential role in defense, possibly acting as natural repellents. Altogether, this multidisciplinary approach underscores the importance of integrating morphological, biochemical, and physiological traits to better understand host-pest interactions in olive orchards. These findings have practical implications for sustainable pest management, suggesting that VOC profiling and cuticle screening could serve as valuable tools for selecting or breeding olive cultivars with enhanced resistance to *B. oleae*. Future research should explore the genetic and molecular mechanisms underlying cuticle biosynthesis and VOC emission, and assess how environmental factors and microbial communities modulate these traits to inform effective, ecologically sound pest control strategies. Understanding the interplay of these factors is essential for developing targeted breeding programs and sustainable management strategies aimed at enhancing resistance against the olive fruit fly.

## Data Availability

The original contributions presented in the study are included in the article/supplementary material. Further inquiries can be directed to the corresponding authors.
